# Supplementary material for: Unmasking coupling between channel gating and ion permeation in the muscle nicotinic receptor
Source: eLife. 2021 Apr 6;10:e66225. doi: 10.7554/eLife.66225 (PMC8024024; doi:10.7554/eLife.66225)
Supplement: Supplementary file 1. — (A) Binding and gating equilibrium constants for the wild-type and salt bridge mutated (DN) receptors. Constants determined by fitting of the open probability data to a sequential bind-bind-gate mechanism. Open probability data and fits shown in Figure 2 in main text. (B) Binding and gating equilibrium constants for β-DN + δ-DN and charge exchanged DKKD receptors. Constants determined by fitting of the open probability data to a sequential bind-bind-gate mechanism. Open probability data and fits shown in Figures 3 and 4 in main text. (C) Average dwell time component weights (W) and time constants (T) of the wild-type muscle receptor at 3, 10, 30, 100, 300 µM [ACh]. Values are averages of three independent recordings with the corresponding standard deviation (SD). (D) Average dwell time component weights (W) and time constants (T) of the β-DN receptor at 3, 10, 30, 100, 300 µM [ACh]. Values are averages of three independent recordings with the corresponding standard deviation (SD). (E) Average dwell time component weights (W) and time constants (T) of the δ-DN receptor at 3, 10, 30, 100, 300 µM [ACh]. Values are averages of three independent recordings with the corresponding standard deviation (SD). (F) Average dwell time component weights (W) and time constants (T) of the beta- + delta- DN receptor at 3, 10, 30, 100, 300 µM [ACh]. Values are averages of three independent recordings with the corresponding standard deviation (SD). (G) Average dwell time component weights (W) and time constants (T) for the δ-DKKD receptor at 3, 10, 30, 100, 300 µM [ACh]. Values are averages of three independent recordings with the corresponding standard deviation (SD). (H) Average dwell time component weights (W) and time constants (T) of the β-DKKD receptor at 3, 10, 30, 100, 300 µM [ACh]. Values are averages of three independent recordings with the corresponding standard deviation (SD). [file elife-66225-supp1.docx]

***Supplementary 1a—Fitted binding and gating constant for WT and DN mutant receptors.***

|  | WT | | | | Beta DN | | | | Delta DN | | | |
| --- | --- | --- | --- | --- | --- | --- | --- | --- | --- | --- | --- | --- |
|  | Value | 95% CI  lower bound | 95% CI  upper bound | Value | | 95% CI  lower bound | 95% CI  upper bound | Value | | 95% CI  lower bound | 95% CI  upper bound |  |
| $\theta$ | 19.2 | 17.51 | 21.19 | 0.1544 | | 0.1474 | 0.1616 | 1.816 | | 1.699 | 1.945 |  |
| $K \mu M$ | 23.14 | 21.6 | 24.86 | 11.15 | | 9.76 | 12.73 | 20.28 | | 18.25 | 22.52 |  |

***Supplementary 1b—Fitted binding and gating constant for double β-DN + δ-DN and charge exchanged DKKD receptors.***

|  | Beta DN + Delta DN | | | Beta DKKD | | | Delta DKKD | | |
| --- | --- | --- | --- | --- | --- | --- | --- | --- | --- |
|  | Value | 95% CI  lower bound | 95% CI  upper bound | Value | 95% CI  lower bound | 95% CI  upper bound | Value | 95% CI  lower bound | 95% CI  upper bound |
| $\theta$ | 0.02164 | 0.01932 | 0.0242 | 3.153 | 2.934 | 3.4 | 9.59 | 8.254 | 11.34 |
| $K \mu M$ | 12.22 | 8.45 | 17.55 | 19.02 | 17.18 | 21.09 | 19.3 | 16.88 | 22.23 |

Fitted gating and binding constants for the wild type and salt bridge mutated (DN) and charge exchanged (DKKKD) receptors. Constants determined by fitting of the open probability data to a sequential bind-bind-gate mechanism. Open probability data and fits shown in Fig 2-4 in main text.

***Supplementary 1c—Fitted parameters for wild type dwell times***

| WT | W1 | SD | T1 (s) | SD (s) | W2 | SD | T2 (s) | SD (s) |
| --- | --- | --- | --- | --- | --- | --- | --- | --- |
| 3 µM Open | 0.153 | 0.063 | 3.48E-04 | 2.42E-04 | 0.847 | 0.063 | 2.64E-03 | 4.65E-04 |
| 3 µM Closed | 0.623 | 0.105 | 2.29E-05 | 6.04E-07 | 0.377 | 0.105 | 1.00E-02 | 2.95E-03 |
| 10 µM Open | 1.000 |  | 1.56E-03 | 8.85E-05 |  |  |  |  |
| 10 µM Closed | 0.517 | 0.002 | 2.81E-05 | 6.15E-06 | 0.483 | 0.002 | 1.41E-03 | 1.15E-04 |
| 30 µM Open | 1.000 |  | 1.52E-03 | 1.41E-04 |  |  |  |  |
| 30 µM Closed | 0.517 | 0.027 | 2.05E-05 | 3.50E-06 | 0.483 | 0.027 | 4.21E-04 | 9.06E-05 |
| 100 µM Open | 1.000 |  | 1.42E-03 | 2.16E-04 |  |  |  |  |
| 100 µM Closed | 0.596 | 0.086 | 1.72E-05 | 9.75E-07 | 0.404 | 0.086 | 1.63E-04 | 1.64E-05 |
| 300 µM Open | 1.000 |  | 9.79E-04 | 9.78E-05 |  |  |  |  |
| 300 µM Closed | 0.796 | 0.006 | 1.50E-05 | 6.11E-07 | 0.204 | 0.006 | 1.25E-04 | 5.75E-06 |

Average dwell time component weights (W) and time constants (T) of the wild type muscle receptor at 3, 10, 30, 100, 300 µM [ACh]. Values are averages of three independent recordings with the corresponding standard deviation (SD).

***Supplementary 1d — Fitted parameters for β-DN dwell times***

| Beta DN | W1 | SD | T1 (s) | SD (s) | W2 | SD | T2 (s) | SD (s) | W3 | SD | T3 (s) | SD (s) |
| --- | --- | --- | --- | --- | --- | --- | --- | --- | --- | --- | --- | --- |
| 3 µM Open | 0.867 | 0.026 | 7.63E-05 | 7.67E-06 | 0.133 | 0.026 | 2.92E-04 | 2.70E-05 |  |  |  |  |
| 3 µM Closed | 0.088 | 0.006 | 5.54E-05 | 1.00E-05 | 0.656 | 0.031 | 2.16E-02 | 3.71E-03 | 0.256 | 0.024 | 9.20E-02 | 2.54E-02 |
| 10 µM Open | 0.900 | 0.054 | 7.22E-05 | 9.85E-06 | 0.100 | 0.054 | 3.71E-04 | 6.66E-05 |  |  |  |  |
| 10 µM Closed | 0.118 | 0.035 | 6.07E-05 | 5.52E-06 | 0.689 | 0.047 | 4.90E-03 | 1.54E-03 | 0.193 | 0.059 | 1.79E-02 | 2.68E-03 |
| 30 µM Open | 0.860 | 0.033 | 7.45E-05 | 2.59E-06 | 0.140 | 0.033 | 2.70E-04 | 2.70E-05 |  |  |  |  |
| 30 µM Closed | 0.066 | 0.005 | 2.81E-05 | 1.44E-05 | 0.656 | 0.089 | 9.82E-04 | 6.57E-05 | 0.279 | 0.090 | 3.77E-03 | 5.52E-04 |
| 100 µM Open | 0.849 | 0.012 | 7.98E-05 | 1.60E-06 | 0.151 | 0.012 | 3.50E-04 | 1.51E-05 |  |  |  |  |
| 100 µM Closed | 0.115 | 0.012 | 7.83E-05 | 1.17E-06 | 0.704 | 0.030 | 6.69E-04 | 3.50E-05 | 0.181 | 0.040 | 2.49E-03 | 4.27E-04 |
| 300 µM Open | 0.852 | 0.077 | 6.18E-05 | 1.26E-05 | 0.146 | 0.073 | 2.16E-04 | 1.15E-05 |  |  |  |  |
| 300 µM Closed | 0.096 | 0.031 | 5.65E-05 | 5.72E-06 | 0.636 | 0.085 | 6.55E-04 | 1.17E-04 | 0.268 | 0.068 | 2.08E-03 | 2.99E-04 |

Average dwell time component weights (W) and time constants (T) of the β-DN receptor at 3, 10, 30, 100, 300 µM [ACh]. Values are averages of three independent recordings with the corresponding standard deviation (SD).

***Supplementary 1e — Fitted parameters of δ-DN dwell times***

| Delta DN | W1 | SD | T1 (s) | SD (s) | W2 | SD | T2 (s) | SD (s) | W3 | SD | T3 (s) | SD (s) |
| --- | --- | --- | --- | --- | --- | --- | --- | --- | --- | --- | --- | --- |
| 3 µM Open | 0.560 | 0.037 | 1.13E-04 | 9.90E-06 | 0.440 | 0.037 | 4.68E-04 | 2.74E-05 |  |  |  |  |
| 3 µM Closed | 0.246 | 0.003 | 3.55E-05 | 4.10E-07 | 0.558 | 0.073 | 1.33E-02 | 1.76E-03 | 0.197 | 0.075 | 4.84E-02 | 1.15E-02 |
| 10 µM Open | 0.610 | 0.032 | 1.26E-04 | 5.18E-06 | 0.390 | 0.032 | 4.07E-04 | 1.27E-05 |  |  |  |  |
| 10 µM Closed | 0.211 | 0.005 | 3.14E-05 | 1.57E-06 | 0.627 | 0.031 | 2.59E-03 | 1.07E-04 | 0.162 | 0.035 | 6.77E-03 | 1.51E-03 |
| 30 µM Open | 0.438 | 0.071 | 1.99E-04 | 2.68E-05 | 0.562 | 0.071 | 6.52E-04 | 3.36E-05 |  |  |  |  |
| 30 µM Closed | 0.197 | 0.034 | 4.34E-05 | 1.80E-05 | 0.663 | 0.095 | 4.86E-04 | 9.58E-05 | 0.140 | 0.072 | 8.22E-04 | 5.27E-04 |
| 100 µM Open | 0.604 | 0.088 | 1.45E-04 | 1.92E-05 | 0.396 | 0.088 | 5.50E-04 | 1.11E-04 |  |  |  |  |
| 100 µM Closed | 0.346 | 0.112 | 1.32E-05 | 1.97E-06 | 0.531 | 0.075 | 2.58E-04 | 7.52E-05 | 0.122 | 0.039 | 1.31E-03 | 4.14E-04 |
| 300 µM Open | 0.608 | 0.035 | 1.71E-04 | 2.72E-06 | 0.392 | 0.035 | 4.60E-04 | 4.57E-05 |  |  |  |  |
| 300 µM Closed | 0.486 | 0.094 | 1.16E-05 | 8.21E-07 | 0.440 | 0.070 | 2.02E-04 | 2.14E-05 | 0.073 | 0.036 | 7.54E-04 | 1.45E-04 |

Average dwell time component weights (W) and time constants (T) of the δ-DN receptor at 3, 10, 30, 100, 300 µM [ACh]. Values are averages of three independent recordings with the corresponding standard deviation (SD).

***Supplementary 1f— Fitted parameters of β-DN + δ-DN dwell times***

| Beta +  Delta DN | W1 | SD | T1 (s) | SD (s) | W2 | SD | T2 (s) | SD (s) | W3 | SD | T3 (s) | SD (s) |
| --- | --- | --- | --- | --- | --- | --- | --- | --- | --- | --- | --- | --- |
| 10 µM Open | 0.874 | 0.085 | 3.66E-05 | 2.49E-06 | 0.118 | 0.100 | 1.97E-04 | 3.78E-05 |  |  |  |  |
| 10 µM Closed | 0.043 | 0.004 | 3.94E-05 | 1.84E-05 | 0.740 | 0.007 | 2.50E-02 | 1.10E-03 | 0.219 | 0.011 | 3.78E-01 | 2.37E-01 |
| 30 µM Open | 0.956 | 0.011 | 2.28E-05 | 3.06E-06 | 0.044 | 0.011 | 2.27E-04 | 3.44E-05 |  |  |  |  |
| 30 µM Closed | 0.077 | 0.030 | 4.73E-05 | 2.69E-05 | 0.484 | 0.170 | 5.10E-03 | 1.38E-03 | 0.439 | 0.171 | 2.10E-02 | 6.79E-03 |
| 100 µM Open | 0.808 | 0.028 | 2.34E-05 | 1.25E-06 | 0.192 | 0.028 | 1.35E-04 | 8.09E-06 |  |  |  |  |
| 100 µM Closed | 0.101 | 0.002 | 4.20E-05 | 9.97E-06 | 0.557 | 0.034 | 1.67E-03 | 1.38E-04 | 0.342 | 0.036 | 7.92E-03 | 7.07E-04 |
| 300 µM Open | 0.931 | 0.009 | 2.44E-05 | 3.52E-06 | 0.069 | 0.010 | 1.26E-04 | 7.66E-06 |  |  |  |  |
| 300 µM Closed | 0.079 | 0.022 | 1.96E-05 | 6.11E-06 | 0.375 | 0.188 | 1.48E-03 | 6.69E-04 | 0.546 | 0.166 | 6.48E-03 | 8.21E-04 |

Average dwell time component weights (W) and time constants (T) of the beta- + delta- DN receptor at 3, 10, 30, 100, 300 µM [ACh]. Values are averages of three independent recordings with the corresponding standard deviation (SD).

***Supplementary 1g — Fitted parameters of δ-DKKD dwell times***

| Delta DKKD | W1 | SD | T1 (s) | SD (s) | W2 | SD | T2 (s) | SD (s) | W3 | SD | T3 (s) | SD (s) |
| --- | --- | --- | --- | --- | --- | --- | --- | --- | --- | --- | --- | --- |
| 3 µM Open | 0.071 | 0.034 | 1.44E-04 | 5.57E-05 | 0.929 | 0.034 | 1.14E-03 | 1.46E-04 |  |  |  |  |
| 3 µM Closed | 0.620 | 0.089 | 3.69E-05 | 1.41E-06 | 0.100 | 0.015 | 1.79E-03 | 1.75E-03 | 0.280 | 0.101 | 1.43E-02 | 1.76E-03 |
| 10 µM Open | 0.146 | 0.052 | 1.73E-04 | 2.22E-05 | 0.854 | 0.052 | 1.04E-03 | 1.93E-05 |  |  |  |  |
| 10 µM Closed | 0.588 | 0.039 | 2.85E-05 | 1.12E-06 | 0.167 | 0.033 | 4.46E-04 | 1.07E-04 | 0.245 | 0.011 | 2.26E-03 | 1.17E-04 |
| 30 µM Open | 0.099 | 0.014 | 1.54E-04 | 6.83E-05 | 0.901 | 0.014 | 1.03E-03 | 1.96E-05 |  |  |  |  |
| 30 µM Closed | 0.705 | 0.050 | 1.18E-05 | 2.46E-06 | 0.153 | 0.005 | 1.10E-04 | 8.19E-06 | 0.142 | 0.045 | 7.41E-04 | 1.80E-05 |
| 100 µM Open | 1.000 |  | 8.60E-04 | 5.11E-05 |  |  |  |  |  |  |  |  |
| 100 µM Closed | 0.747 | 0.003 | 9.11E-06 | 6.27E-07 | 0.180 | 0.015 | 9.43E-05 | 1.34E-05 | 0.072 | 0.016 | 4.75E-04 | 1.77E-04 |
| 300 µM Open | 1.000 |  | 6.30E-04 | 4.47E-05 |  |  |  |  |  |  |  |  |
| 300 µM Closed | 0.871 | 0.024 | 9.26E-06 | 1.78E-06 | 0.105 | 0.018 | 3.66E-05 | 2.03E-05 | 0.024 | 0.007 | 3.01E-04 | 6.29E-05 |

Average dwell time component weights (W) and time constants (T) for the δ-DKKD receptor at 3, 10, 30, 100, 300 µM [ACh]. Values are averages of three independent recordings with the corresponding standard deviation (SD).

***Supplementary 1h— Fitted parameters of β-DKKD dwell times***

| Beta DKKD | W1 | SD | T1 (s) | SD (s) | W2 | SD | T2 (s) | SD (s) | W3 | SD | T3 (s) | SD (s) |
| --- | --- | --- | --- | --- | --- | --- | --- | --- | --- | --- | --- | --- |
| 3 µM Open | 0.664 | 0.109 | 2.59E-04 | 7.94E-05 | 0.336 | 0.110 | 9.39E-04 | 4.41E-04 |  |  |  |  |
| 3 µM Closed | 0.329 | 0.072 | 3.60E-05 | 9.17E-07 | 0.193 | 0.066 | 3.31E-03 | 1.69E-03 | 0.478 | 0.017 | 1.54E-02 | 3.90E-03 |
| 10 µM Open | 0.421 | 0.077 | 1.52E-04 | 2.38E-05 | 0.579 | 0.077 | 8.37E-04 | 1.70E-04 |  |  |  |  |
| 10 µM Closed | 0.369 | 0.086 | 3.41E-05 | 1.43E-05 | 0.198 | 0.092 | 7.11E-04 | 2.83E-04 | 0.458 | 0.106 | 2.80E-03 | 6.42E-04 |
| 30 µM Open | 0.382 | 0.036 | 1.28E-04 | 1.65E-05 | 0.618 | 0.000 | 5.87E-04 | 6.91E-05 |  |  |  |  |
| 30 µM Closed | 0.316 | 0.008 | 2.42E-05 | 1.77E-06 | 0.632 | 0.018 | 3.82E-04 | 1.03E-05 | 0.052 | 0.011 | 1.59E-03 | 5.12E-04 |
| 100 µM Open | 0.339 | 0.073 | 1.13E-04 | 2.20E-05 | 0.661 | 0.073 | 5.01E-04 | 8.75E-05 |  |  |  |  |
| 100 µM Closed | 0.295 | 0.025 | 2.12E-05 | 4.03E-06 | 0.569 | 0.072 | 2.01E-04 | 4.86E-05 | 0.136 | 0.075 | 7.06E-04 | 2.12E-04 |
| 300 µM Open | 0.336 | 0.037 | 1.78E-04 | 1.51E-05 | 0.664 | 0.037 | 6.57E-04 | 2.66E-05 |  |  |  |  |
| 300 µM Closed | 0.240 | 0.089 | 2.24E-05 | 1.51E-05 | 0.633 | 0.060 | 1.73E-04 | 1.85E-05 | 0.128 | 0.061 | 5.49E-04 | 3.60E-05 |

Average dwell time component weights (W) and time constants (T) of the β-DKKD receptor at 3, 10, 30, 100, 300 µM [ACh]. Values are averages of three independent recordings with the corresponding standard deviation (SD).
