# Supplementary material for: Unmasking coupling between channel gating and ion permeation in the muscle nicotinic receptor
Source: eLife. 2021 Apr 6;10:e66225. doi: 10.7554/eLife.66225 (PMC8024024; doi:10.7554/eLife.66225)
Supplement: Supplementary file 2. — (A) Frequency and magnitude of open channel current fluctuations in wild type and DN receptors. Ss, Sf, fcs, fcf, Sc determined from least squares fit of Equation 1 in main text to open channel current power spectra. SD/A indicates open channel current standard deviation relative to channel amplitude. Spread is shown as 95% confidence or standard deviation as indicated in table. Fits obtained from the average spectrum of at least 50 open channel segments with baseline spectra subtracted. (B) Power spectra and open channel current relaxations of the β-DN + δ-DN receptor. Best fit parameters obtained from simultaneous least squares fit of Equation 1 from main text, Equations A7 and A8 to the power spectrum and open channel current relaxations for the β-DN + δ-DN receptor. [file elife-66225-supp2.docx]

***Supplementary File 2a—Frequency and magnitude of open channel current fluctuations in wild type and DN receptors***

|  | $S_{s}$  (A^2^/Hz) | 95% CI  (A^2^/Hz) | $f_{cs}$ (Hz) | 95% CI  (Hz) | $S_{f}$  (A^2^/Hz) | 95% CI  (A^2^/Hz) | $f_{cf}$ (Hz) | 95% CI  (Hz) | $S_{c}$  (A^2^/Hz) | 95% CI  (A^2^/Hz) | SD/A | SD |
| --- | --- | --- | --- | --- | --- | --- | --- | --- | --- | --- | --- | --- |
| WT |  |  |  |  | 6.17E-29 | 5.75E-29 to 6.76E-29 | 1935 | 1732 to 2138 | 1.74E-29 | 1.62E-29 to 1.82E-29 | 0.041 | 0.0043 |
| β DN | 2.24E-28 | 1.86E-28 to  2.69E-28 | 416.2 | 289.8 to  542.7 | 1.05E-28 | 9.12E-29 to  1.20E-28 | 4173 | 3239 to  5108 | 5.13E-29 | 4.47E-29 to  5.89E-29 | 0.096 | 0.0105 |
| β + δ DN | 1.58E-27 | 1.26E-27 to  2.04E-27 | 113.5 | 86.54 to  140.4 | 4.27E-28 | 3.98E-28 to  4.57E-28 | 2842 | 2612 to  3072 | 1.12E-28 | 1.05E-28 to  1.17E-28 | 0.159 | 0.0173 |

$S_{s}$, $S_{f}$, $f_{cs}$, $f_{cf}$, $S_{c}$ determined from least squares fit of Eq. 1 in main text to open channel current power spectra. SD/A indicates open channel current standard deviation relative to channel amplitude. Spread is shown as 95% confidence or standard deviation as indicated in table. Fits obtained from the average of spectrum of at least 50 open channel segments with baseline spectra subtracted.

***Supplementary File 2b—Power spectra and open channel current relaxations of the β-DN + δ-DN receptor***

|  | $f_{cs}$  (Hz) | $f_{cf}$  (Hz) | $A_{\alpha s}$  O→C | $A_{\alpha f}$  O→C | $A_{\beta s}$  C→O | $A_{\beta f}$  C→O | $S_{s}$  (A^2^/Hz) | $S_{f}$  (A^2^/Hz) | $S_{c}$  (A^2^/Hz) |
| --- | --- | --- | --- | --- | --- | --- | --- | --- | --- |
| Value | 118.34 | 2852.9 | -0.0053 | -0.0401 | 0.0278 | 0.0728 | 1.55E-27 | 4.25E-28 | 1.11E-28 |
| 95% CI | 125.61 to  111.07 | 2790.9 to  2914.8 | -0.0083 to  -0.0022 | -0.0545 to  -0.0257 | 0.0247 to  0.031 | 0.0583 to  0.0872 | 1.46E-27 to  1.64E-27 | 4.18E-28 to  4.318E-28 | 1.09E-28 to  1.13E-28 |

Parameters obtained from simultaneous least squares fit of Eq. 1 from main text, Eq. A7, and Eq. A8 to the power spectra and open channel current relaxations for the β-DN + δ-DN receptor.
